# Dietary fat intake and risk of disabling hearing impairment: a prospective population-based cohort study

**DOI:** 10.1007/s00394-021-02644-7

**Published:** 2021-07-21

**Authors:** Humberto Yévenes-Briones, Francisco Félix Caballero, Ellen A. Struijk, Alberto Lana, Fernando Rodríguez-Artalejo, Esther Lopez-Garcia

**Affiliations:** 1 Department of Preventive Medicine and Public Health, School of Medicine, Universidad Autónoma de Madrid-IdiPaz and CIBERESP (CIBER of Epidemiology and Public Health), C/ Arzobispo Morcillo, s/n, 28029 Madrid, Spain; 2 Department of Medicine, School of Medicine and Health Sciences, Universidad de Oviedo /ISPA, Oviedo, Spain; 3 IMDEA-Food Institute, CEI UAM+CSIC, Madrid, Spain

**Keywords:** Dietary fat intake, Hearing function, UK Biobank, Cohort study

## Abstract

**Purpose:**

To examine the associations of specific dietary fats with the risk of disabling hearing impairment in the UK Biobank study.

**Methods:**

This cohort study investigated 105,592 participants (47,308 men and 58,284 women) aged ≥ 40 years. Participants completed a minimum of one valid 24-h recall (Oxford Web-Q). Dietary intake of total fatty acids, polyunsaturated fatty acids (PUFA), saturated fatty acids (SFA), and monounsaturated fatty acids (MUFA) was assessed at baseline. Functional auditory capacity was measured with a digit triplet test (DTT), and disabling hearing impairment was defined as a speech reception threshold in noise > − 3.5 dB in any physical exam performed during the follow-up.

**Results:**

Over a median follow-up of 3.2 (SD: 2.1) years, 832 men and 872 women developed disabling hearing impairment. After adjustment for potential confounders, including lifestyles, exposure to high-intensity sounds, ototoxic medication and comorbidity, the hazard ratios (HRs), and 95% confidence interval (CI) of disabling hearing function, comparing extreme quintiles of intakes were 0.91 (0.71–1.17) for total fat, 1.09 (0.83–1.44) for PUFA, 0.85 (0.64–1.13) for SFA and 1.01 (0.74–1.36) for MUFA among men. Among women, HRs comparing extreme intakes were 0.98 (0.78–1.24) for total fat, 0.69 (0.53–0.91) for PUFA, 1.26 (0.96–1.65) for SFA, and 0.91 (0.68–1.23) for MUFA. Replacing 5% of energy intake from SFA with an equivalent energy from PUFA was associated with 25% risk reduction (HR: 0.75; 95% CI: 0.74–0.77) among women.

**Conclusions:**

PUFA intake was associated with decreased risk of disabling hearing function in women, but not in men.

**Supplementary Information:**

The online version contains supplementary material available at 10.1007/s00394-021-02644-7.

## Introduction

Hearing loss is one of the main leading causes of years lived with disability; moreover, it has been considered an “invisible disability”, since it is usually underestimated in comparison with other health problems [1]. In older people, hearing loss has been associated with higher risk of social isolation [2], depression [3], cognitive impairment [4], poor quality of life [5], and also with higher risk of cardiovascular disease and all-cause mortality [6–8]. In addition, a large body of evidence suggests that sex modulates susceptibility to age-related hearing loss, because of the protective effect of estrogens on hearing function and differences in the processing of stimuli at the cortical level [9].

Besides age-associated biological degeneration and noise exposure, one mechanism of hearing loss is impaired vascular function. The cochlea of the inner ear is highly vascularized and supported by a single artery; thus, dietary exposures that are able to improve vascular function may have a protective role on hearing capacity. However, the evidence on the effect of habitual diet on hearing loss is limited [10, 11]. High intake of some nutrients, such as β-carotene, β-cryptoxanthin, vitamin B12, folic acid, vitamin D, and magnesium, have been associated with lower risk of hearing loss [12–15]. As regards macronutrient intake, three studies have focused on the effect of polyunsaturated fatty acids (PUFA). They found that a higher intake of n-3 PUFA was associated with decreased hearing loss risk [16–18]. In addition, a cross-sectional study found that low fat and protein intakes were associated with hearing impairment [19].

There is compelling evidence that different types of dietary fats have opposed effects on cardiovascular disease by modifying serum lipid profiles, endothelial function, chronic inflammation, and blood clotting, and that the type of fat is more important than the total amount ingested [20]. Similarly, different associations have been found between distinct types of dietary fats and risk of frailty [21] or physical function impairment [22]. Understanding the effect of habitual intake of specific fatty acids on hearing function could help to develop dietary recommendations for healthy aging, including optimal hearing. Therefore, the objective of this study was to examine the prospective association of fatty acids intake with disabling hearing impairment, assessed through the functional auditory capacity, in a large population-based study of middle and older-age men and women of the UK.

## Methods

### Study design and participants

The UK Biobank study is a large population-based cohort study established in 2006–2010 throughout the United Kingdom [23]. The study recruited more than 500,000 participants aged between 40 and 70 years, who gave information on health status, demographics, and lifestyle. In addition, they provided several types of biological samples and underwent a physical examination. Participants were followed to update information in 2012–2013 and in 2014–2016.

### Dietary assessment

Food consumption was collected with five 24-h recalls (Oxford Web-Q), a detailed computerized questionnaire on the intake of 200 commonly consumed foods and beverages in the previous 24 h [24, 25]. Unlike standard 24-h diet recalls, where the respondents are asked to remember and report the food consumed, the Web-Q presents 21 food groups and asks the participants if they consumed any of them over the previous day. Positive answers open additional questions in which participants have to select the type of food consumed and its amount, based on standard serving categories or portions. Thus, the data collection approach used in this tool could be defined as a hybrid between a 24-h dietary recall and a food frequency questionnaire [24]. The Web-Q automatically calculated nutrient intakes from food composition tables specific for the United Kingdom [26]. Since we focused on average macronutrient intakes, which are stable to day-to-day variation on food consumption [27], we included those participants who completed at least one Web-Q, and calculated the average nutrient intake from all available Web-Qs. For the calculation of monounsaturated fatty acids (MUFA), we subtracted saturated (SFA) and PUFA from the total fat intake [28]. Intake of subtypes of PUFA, trans fat, and dietary cholesterol were not available. Total and specific fatty acids intake was expressed as a percentage of ingested energy. In a recent publication, main sources of total fats have been identified as the following food groups: “desserts and cakes and pastries”, “high fat cheese”, “dairy fat spread”, “egg and egg dishes”, and “biscuits”; main sources of SFA were “high fat cheese”, “desserts and cakes and pastries”, “dairy fat spread”, “milk-dairy desserts”, and “biscuits”. No information of main sources of PUFA was available [29]. Finally, overall diet quality was assessed by adherence to the alternate Mediterranean Diet score (aMED), after excluding the component score for fatty acids [30].

### Functional auditory capacity and hearing-related variables

Functional auditory capacity was measured with a digit triplet test (DTT) to determine the speech reception threshold noise (SRTn). The SRTn is a measure of the ability to understand speech in noise. Before starting the test, participants were asked to remove their hearing aid if they had it. In addition, the volume of the speech was set to the individual’s most comfortable level for each ear. Then, the participant listened to 15 sets of three digits presented with background noise and had to enter each triplet on a keyboard on the touch screen. If the triplet was correctly identified, the noise level was increased for the next triplet; otherwise, the noise level was decreased. Each ear was tested separately, and SRTn was defined as the signal-to-noise-ratio at which half of the presented digits could be recognized correctly. The signal-to-noise-ratio could range between − 12 and + 8 dB. In the analyses, we used the SRTn for the best ear in each participant in both measurements, at baseline and at the follow-up, and if the SRTn was only available for one ear, we assumed that it was the best one. Dawes et al. [31] have established the cut-off points to categorize the UK Biobank population as with normal (SRTn < − 5.5 dB), insufficient (SRTn ≥ − 5.5 to ≤ − 3.5 dB) or poor hearing function (SRTn > − 3.5 dB). We defined disabling hearing impairment as a SRTn > − 3.5 dB in any physical exam during the follow-up. The DTT has shown a good correlation with pure-tone audiometry (*r* = 0.77), which suggests that about 60% of the performance on DTT is explained by standardized audiometric data [32]. The differences in the psychoacoustic ability of the listeners influence the ability to recognize speech in noise, which explains the remaining variation [33].

Several hearing-related variables were also obtained. Loud music exposure, noisy workplace, and tinnitus were, respectively, assessed by asking the participants: “Have you ever listened to music for more than 3 h per week at a volume which you would need to shout to be heard or, if wearing headphones, someone else would need to shout for you to hear them?”; “Have you ever worked in a noisy place where you had to shout to be heard?”; and “Do you get or have you had noises (such as ringing or buzzing) in your head or in one or both ears that lasts for more than five minutes at a time?” [34].

### Mortality

All-cause mortality was obtained from death certificates held by the National Health Service Information Centre (England and Wales) and the National Health Service Central Register Scotland (Scotland) [35].

### Other variables

Baseline information included age, sex, ethnicity, educational level, and smoking status. Weight and height were also measured under standardized conditions, and body mass index (BMI) was calculated as weight (kg) divided by height (m) squared. Physical activity (metabolic equivalent tasks-hours/week, METs-h/wk) was evaluated with the Short International Physical Activity Questionnaire [36]. Cognitive function was assessed through the reaction time test, by showing 12 rounds of pairs of cards to each participant, who had to press a button as quickly as possible if both cards were the same. The test allows to calculate the average reaction time (milliseconds) of each participant to identify the pairs of cards; a longer time indicated a worse cognitive status [37]. Finally, diagnoses of diabetes, vascular or heart problems, hypercholesterolemia, or cancer and use of ototoxic medication were reported by the participants.

### Statistical analysis

On a sample of 211,013 participants with dietary information, we excluded 1,023 with unrealistic energy intake (< 800 or > 5,000 kcal/day for men, and < 500 or > 4000 kcal/day for women), 103,075 without a hearing test at baseline, and 1323 with disabling hearing at baseline, leaving a total of 105,592 participants for the analysis (47,308 men and 58,284 women).

Participants were classified into quintiles of percentage of energy from total fat, PUFA, SFA, and MUFA. We used the analysis of variance (ANOVA) or Chi-square test, depending on the type of variable (continuous or categorical ones, respectively), to assess differences in sociodemographic characteristics, lifestyle, and morbidity across the quintiles of total fatty acid intake.

Person-years of follow-up were calculated from the first interview at baseline until the occurrence of disabling hearing impairment, death, loss to follow-up, or the end of the study (December 2016), whichever came first. We used Cox proportional hazard models to calculate hazard ratios (HRs), and their 95% confidence intervals (CI), for the association between each quintile of total fat and PUFA, SFA, and MUFA intake and disabling hearing impairment, adjusting for potential confounders. Two types of models were built. The first model was adjusted for age. A second model was additionally adjusted for ethnic background (British/other), educational level (primary education or less, secondary education, and university degree), tobacco consumption (current smoker, former smoker, never smoker), BMI (< 25.0, 25.0–29.9, ≥ 30.0 kg/m^2^), physical activity (quintiles of METs hour/week), alcohol consumption (quintiles of g/d), loud music exposure (yes/no), noisy workplace (no, for less than one year, for around 1–5 years, for more than 5 years), presence of tinnitus, aspirin and ibuprofen consumption (yes/no), reaction time in cognitive function test (ms), hypercholesterolemia, vascular or heart problems, cancer, diabetes, total energy (quintiles of kcal/day) and protein intake, and the other fatty acids intake (quintiles of % energy), as appropriate. Thus, the coefficient for dietary fat reflects the effect of substituting an equal amount of energy from fat for carbohydrate. Tests for linear trend were conducted by assigning the median value to each quintile and treating this as a continuous variable in the regression models. To test nonlinear risk trends, we used three knot restricted cubic splines for the consumption of specific fatty acids and the risk of disabling hearing function [38].

We performed separate analyses in women and men, as we found a statistically significant interaction term for sex and intake of PUFA when predicting incident hearing impairment (*P* = 0.03). Also, and according to the previous literature, we performed analyses stratified by subgroups of age [39], presence of tinnitus [40], being overweight or obese [41], having chronic diseases [42], and diet quality [30]. Additionally, we conducted separate analyses among those with optimal hearing at the start of the study, to understand whether the effect of fatty acids depends on the baseline hearing status. Finally, we evaluated the effect of substituting one type of dietary fat for an equal amount of another type of fat on hearing impairment. To fit these isocaloric energy density models, we simultaneously included total energy intake and the percentage of energy derived from the types of fats of interest as continuous variables (per 5% increase), along with the covariates listed above, and calculated the difference in coefficients. Additionally, we modeled the substitution of each type of dietary fat for carbohydrates and for total protein.

Analyses were performed with Stata (version 15.0; Stata Corp., College Station). This manuscript follows the Strengthening the Reporting of Observational Studies in Epidemiology (STROBE) recommendations.

## Results

In this cohort, mean (SD) intakes of fatty acids were 14.3 (29.8) g/d for PUFA, 29.5 (12.8) g/d for SFA, and 33.2 (13.2) g/d for MUFA. The percentages of energy provided for each specific fat were 6.1, 12.5, and 14.1%, respectively. Baseline characteristics of the population by quintiles of total fat and sex are presented in Table 1. Compared to participants in the lowest quintile of total fat intake, those in the highest quintile had lower prevalence of primary or less education, were more likely to smoke, reported lower physical activity, were more likely exposed to loud music, and had higher cognitive performance. Ibuprofen consumption was higher among those with the highest intake, but hypercholesterolemia and vascular or heart problems were less prevalent in that group. Protein and carbohydrate intakes decreased across quintiles of increased fat intake.

**Table 1:** Participants’ characteristics at baseline across the quintiles of total fat intake (% energy) in the UK Biobank study by sex (*N* = 105,592)

	Quintiles of total fat intake, % energy									
	Men (*n* = 47,308)					Women (*n* = 58,284)				
	Q1	Q2	Q3	Q4	Q5	Q1	Q2	Q3	Q4	Q5
Participants, *n*	9462	9462	9461	9462	9461	11,657	11,657	11,657	11,657	11,656
Age, y	57.0 ± 8.1	57.2 ± 8.1	57.2 ± 8.1	56.7 ± 8.1	56.0 ± 8.3^***^	56.4 ± 7.8	56.3 ± 7.9	56.0 ± 8.0	55.9 ± 8.0	55.3 ± 8.1^***^
British, %	84.9	87.9	88.9	90.0	88.4^***^	85.0	86.7	86.8	87.2	85.4^***^
Educational level ≤ primary, %	11.1	9.2	9.8	9.7	10.5^***^	11.2	9.7	8.8	9.1	9.9^***^
Current smoker, %	9.7	8.4	9.2	9.5	12.1^***^	6.6	5.8	6.2	7.2	9.3^***^
BMI, Kg/m^2^	27.6 ± 3.9	27.5 ± 4.0	27.5 ± 4.1	27.6 ± 4.2	27.9 ± 4.4^***^	26.7 ± 5.0	26.6 ± 4.9	26.5 ± 4.9	26.7 ± 5.1	27.1 ± 5.5^***^
Physical activity, METs-h/wk	45.0 ± 44.5	44.2 ± 42.9	43.0 ± 42.7	41.8 ± 42.1	41.2 ± 42.2^***^	42.6 ± 38.3	41.4 ± 37.1	40.4 ± 36.9	38.8 ± 35.1	39.0 ± 36.5^***^
Loud music exposure, %	17.5	17.8	17.1	18.4	20.4^***^	8.9	8.9	9.0	9.6	11.1^***^
Noisy workplace, %	32.4	32.2	32.2	32.2	33.2	10.0	8.9	8.5	9.4	10.1^***^
Tinnitus, %	18.9	19.9	20.3	19.5	20.2	14.1	14.8	13.9	14.6	14.7
Aspirin consumption, %	17.6	16.0	15.6	15.2	15.2^***^	7.6	7.5	7.5	7.8	7.8
Ibuprofen consumption, %	11.5	11.8	11.7	12.6	12.8^*^	17.4	17.2	18.2	18.2	18.8^***^
Poor cognitive performance^a^, %	12.5	12.1	11.5	11.4	11.5	15.3	14.5	13.9	14.2	13.5^**^
Hypercholesterolemia, %	26.2	23.6	23.2	21.1	21.0^***^	13.0	11.7	10.8	10.2	10.8^***^
Vascular/heart problems^b^, %	35.6	33.7	32.6	31.4	31.1^***^	25.0	22.9	21.7	22.7	22.4^***^
Cancer, %	6.1	6.5	6.3	6.2	6.3	8.8	9.3	9.0	9.5	9.0
Diabetes, %	6.5	5.8	6.0	6.4	7.6^***^	3.1	3.1	3.1	2.9	4.0^***^
Dietary intake										
Total energy intake, kcal/d	2055 ± 618	2232 ± 607	2310 ± 602	2391 ± 628	2432 ± 683^***^	1745 ± 522	1892 ± 492	1966 ± 493	2030 ± 507	2057 ± 561^***^
Total fat intake, % of energy	22.6 ± 3.6	28.9 ± 1.2	32.5 ± 1.0	36.0 ± 1.1	41.9 ± 3.5^**^	23.2 ± 3.6	29.4 ± 1.2	33.0 ± 1.0	36.5 ± 1.1	42.7 ± 3.8^***^
PUFA intake, % of energy	4.1 ± 1.6	5.3 ± 1.7	6.0 ± 1.9	6.6 ± 2.0	7.6 ± 2.4^***^	4.3 ± 1.6	5.5 ± 1.8	6.2 ± 1.9	6.9 ± 2.1	8.0 ± 2.6^***^
SFA intake, % of energy	8.7 ± 2.3	11.2 ± 2.1	12.5 ± 2.2	14.0 ± 2.3	16.1 ± 3.1^***^	8.9 ± 2.4	11.3 ± 2.2	12.6 ± 2.2	13.9 ± 2.4	16.0 ± 3.2
MUFA intake, % of energy	9.8 ± 2.0	12.4 ± 1.3	14.0 ± 1.3	15.5 ± 1.4	18.2 ± 2.3^***^	10.0 ± 2.0	12.7 ± 1.4	14.2 ± 1.4	15.8 ± 1.5	18.7 ± 2.6^***^
Protein intake, % of energy	15.6 ± 4.1	15.6 ± 3.4	15.5 ± 3.3	15.4 ± 3.4	15.4 ± 3.7^***^	17.0 ± 4.3	16.6 ± 3.7	16.2 ± 3.5	15.9 ± 3.4	15.7 ± 4.0^***^
Carbohydrate intake, % of energy	53.1 ± 10.4	50.3 ± 7.7	48.2 ± 6.9	46.1 ± 6.2	41.6 ± 6.7^***^	56.3 ± 8.8	51.7 ± 6.7	49.2 ± 6.0	46.5 ± 5.7	41.3 ± 6.7^***^
Alcohol intake, g/d	33.5 ± 33.9	25.0 ± 26.0	21.3 ± 23.4	17.4 ± 20.5	12.4 ± 17.6^***^	15.3 ± 20.1	13.0 ± 16.8	11.6 ± 15.1	10.4 ± 13.9	7.8 ± 12.2^***^

*BMI* body mass index, *METs* metabolic equivalent tasks, *PUFA* polyunsaturated fatty acids, *SFA* saturated fatty acids, *MUFA* monounsaturated fatty acids

Values are means ± SD unless otherwise indicated

*P* values based on ANOVA test for continuous variables or Chi-square test for qualitative variables

*P < 0.05 ^**^P < 0.01 ^***^P < 0.001

^a^Defined as reaction time (ms) greater than the mean plus a standard deviation

^b^Includes heart attack, angina, stroke, and hypertension

Over a median follow-up of 3.2 years, 1704 (1.61%) participants developed disabling hearing impairment [832 (1.76%) men and 872 (1.50%) women]. Among men, in fully adjusted models, no significant associations were found between fatty acids intake and hearing impairment: HR (95% CI) for quintile 5 vs. 1 were 0.91 (0.71–1.17) for total fat; 1.09 (0.83–1.44) for PUFA; 0.85 (0.64–1.13) for SFA; and 1.01 (0.74–1.36) for MUFA (Table 2). Among women, although no association was observed between total fat intake and disabling hearing impairment, an inverse association was found for PUFA, (HR quintile 5 vs. 1: 0.69; 95% CI: 0.53–0.91). On the contrary, SFA and MUFA intakes were not associated with the outcome (Table 2).

**Table 2:** Association between dietary fat intake (% energy) and risk of disabling hearing function in the UK Biobank study by sex (*N* = 105,592)

	Quintile 1	Quintile 2	Quintile 3	Quintile 4	Quintile 5	*P* trend
Men						
Total fat intake						
Participants, *n*	9462	9462	9461	9462	9461	
Range, % energy	1.6–26.7	26.8–30.7	30.8–34.1	34.2–37.9	38.0–68.6	
Person-yr	36,053	37,404	37,557	37,400	36,815	
Cases, *n*	151	184	196	159	142	
Age-adjusted model	1.00	1.01 (0.82–1.26)	1.07 (0.87–1.33)	0.90 (0.72.1.12)	0.89 (0.70–1.11)	0.17
MV-adjusted model	1.00	1.04 (0.83–1.29)	1.11 (0.89–1.39)	0.94 (0.74–1.19)	0.91 (0.71–1.17)	0.31
PUFA intake						
Participants, *n*	9462	9462	9461	9462	9461	
Range, % energy	0.8–3.9	4.0–5.1	5.2–6.2	6.3–7.6	7.7–18.7	
Person-yr	35,766	37,610	37,642	37,612	36,599	
Cases, *n*	130	179	180	198	145	
Age-adjusted model	1.00	1.09 (0.87–1.37)	1.12 (0.89–1.40)	1.22 (0.98–1.52)	1.07 (0.84–1.35)	0.45
MV-adjusted model	1.00	1.11 (0.88–1.40)	1.15 (0.90–1.46)	1.24 (0.97–1.59)	1.09 (0.83–1.44)	0.48
SFA intake						
Participants, *n*	9462	9462	9461	9462	9461	
Range, % energy	0.3–9.5	9.6–11.4	11.5–13.1	13.2–15.2	15.3–30.5	
Person-yr	36,419	37,134	37,558	37,432	36,686	
Cases, *n*	166	185	171	173	137	
Age-adjusted model	1.00	1.01 (0.82–1.25)	0.89 (0.72–1.10)	0.93 (0.75–1.15)	0.81 (0.65–1.02)	0.05
MV-adjusted model	1.00	1.04 (0.83–1.29)	0.91 (0.72–1.15)	0.97 (0.76–1.24)	0.85 (0.64–1.13)	0.23
MUFA intake						
Participants, *n*	9462	9462	9461	9462	9461	
Range, % energy	0.6–11.3	11.4–13.1	13.2–14.7	14.8–16.5	16.6–37.0	
Person-yr	35,927	37,412	37,481	37,414	36,995	
Cases, *n*	145	187	180	161	159	
Age-adjusted model	1.00	1.05 (0.84–1.30)	1.00 (0.80–1.25)	0.93 (0.74–1.16)	0.97 (0.77–1.21)	0.48
MV-adjusted model	1.00	1.03 (0.81–1.30)	1.02 (0.79–1.31)	0.95 (0.72–1.26)	1.01 (0.74–1.36)	0.87
Women						
Total fat intake						
Participants, *n*	11,657	11,657	11,657	11,657	11,656	
Range, % energy	1.6–27.2	27.3–31.2	31.3–34.6	34.7–38.5	38.6–70.9	
Person-yr	43,174	44,272	44,430	44,633	43,703	
Cases, *n*	152	185	186	187	162	
Age-adjusted model	1.00	1.05 (0.85–1.30)	1.04 (0.84–1.29)	1.04 (0.84–1.29)	1.02 (0.82–1.27)	0.90
MV-adjusted model	1.00	1.04 (0.84–1.30)	1.02 (0.82–1.27)	1.01 (0.81–1.26)	0.98 (0.78–1.24)	0.79
PUFA intake						
Participants, *n*	11,657	11,657	11,657	11,657	11,656	
Range, % energy	0.03–4.0	4.1–5.2	5.3–6.4	6.5–7.9	8.0–25.3	
Person-yr	42,908	44,465	44,530	44,370	43,939	
Cases, *n*	168	184	183	204	133	
Age-adjusted model	1.00	0.89 (0.72–1.09)	0.88 (0.71–1.08)	1.01 (0.82–1.24)	0.69 (0.55–0.87)	0.01
MV-adjusted model	1.00	0.86 (0.69–1.07)	0.84 (0.67–1.06)	0.98 (0.77–1.23)	0.69 (0.53–0.91)	0.04
SFA intake						
Participants, *n*	11,657	11,657	11,657	11,657	11,656	
Range, % energy	0.1–9.6	9.7–11.5	11.6–13.2	13.3–15.2	15.3–33.8	
Person-yr	42,963	44,328	44,530	44,532	43,859	
Cases, *n*	127	201	187	182	175	
Age-adjusted model	1.00	1.34 (1.07–1.67)	1.23 (0.99–1.55)	1.22 (0.97–1.53)	1.27 (1.01–1.59)	0.17
MV-adjusted model	1.00	1.36 (1.08–1.71)	1.24 (0.97–1.58)	1.19 (0.93–1.53)	1.26 (0.96–1.65)	0.36
MUFA intake						
Participants, *n*	11,657	11,657	11,657	11,657	11,656	
Range, % energy	0.8–11.5	11.6–13.3	13.4–14.9	15.0–16.8	16.9–38.7	
Person-yr	43,137	44,325	44,656	44,551	43,543	
Cases, *n*	157	182	202	190	144	
Age-adjusted model	1.00	1.03 (0.83–1.27)	1.09 (0.88–1.34)	1.04 (0.84–1.29)	0.90 (0.72–1.13)	0.42
MV-adjusted model	1.00	1.04 (0.82–1.31)	1.09 (0.85–1.38)	1.03 (0.79–1.33)	0.91 (0.68–1.23)	0.51

*PUFA* polyunsaturated fatty acids, *SFA* saturated fatty acids, *MUFA* monounsaturated fatty acids

Values are hazard ratios (95% confidence interval)

Multivariable (MV) adjusted model: Cox regression model adjusted for age, ethnic background, educational level (≤ primary, secondary, university), tobacco (current smoker, former smoker, never smoker), BMI (< 25.0, 25.0–29.9, ≥ 30.0 kg/m^2^), physical activity (quintiles of METs-h/wk), alcohol consumption (quintiles of g/d), loud music exposure (yes/no), noisy workplace (no, for less than a year, for around 1–5 years, for more than 5 years), tinnitus, aspirin, ibuprofen consumption, reaction time (ms), hypercholesterolemia, vascular/heart problems, cancer, diabetes, total energy (quintiles of kcal/day), and protein intake (quintiles of % energy). Models for PUFA were adjusted for SFA and MUFA and vice versa (quintiles of % energy)

**Fig. 1:**
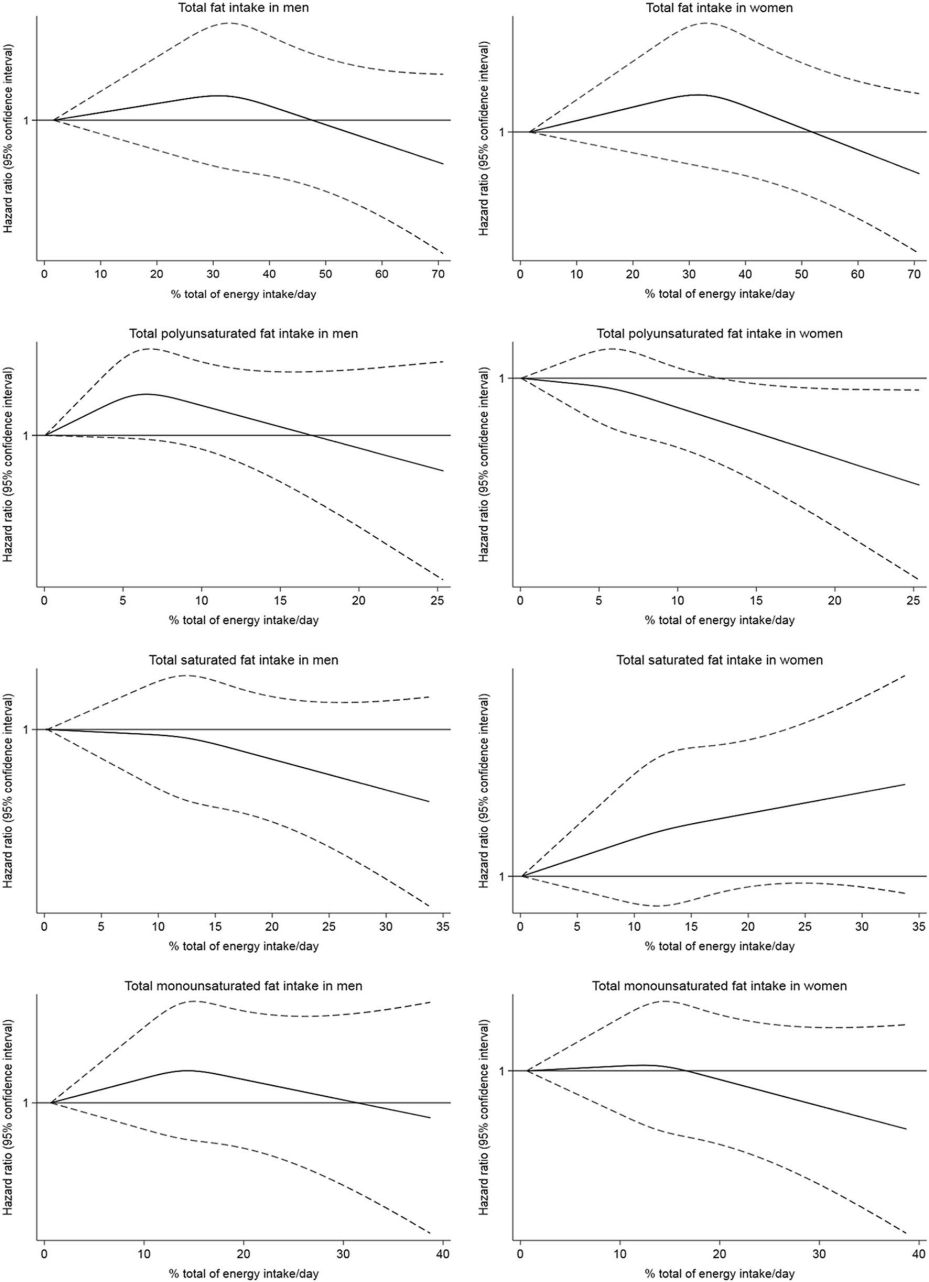
Multivariable adjusted spline curves for the relation between dietary fat intake and the risk of disabling hearing function in men and women. Covariates were age, ethnic background, educational level (≤ primary, secondary, university), tobacco (current smoker, former smoker, never smoker), BMI (< 25.0, 25.0–29.9, ≥ 30.0 kg/m^2^), physical activity (quintiles of METs-h/wk), alcohol consumption (quintiles g/d), loud music exposure (yes/no), noisy workplace (no, for less than a year, for around 1–5 years, for more than 5 years), tinnitus, aspirin, ibuprofen consumption, reaction time (ms), hypercholesterolemia, vascular/heart problems, cancer, diabetes, total energy (quintiles of kcal/day), and protein intake (quintiles of % energy), and for PUFA, SFA, and MUFA (in quintiles of % energy), as appropriate

The dose–response association between the fatty acids and disabling hearing impairment was assessed in Fig. 1. For PUFA, an inverse association was found in women, becoming statistically significant with intakes ≥ 15% of energy. We estimated that replacing 5% of energy intake from SFA with an equivalent energy from PUFA was associated with 25% risk reduction (HR: 0.75; 95% CI: 0.74–0.77). Replacing 5% of energy intake from MUFA for PUFA marginally reduced the risk (HR: 0.97; 95% CI: 0.94–1.00), while substitution of carbohydrates and total protein for PUFAs also showed a risk reduction (Supplemental Table 1).

**Fig. 2:**
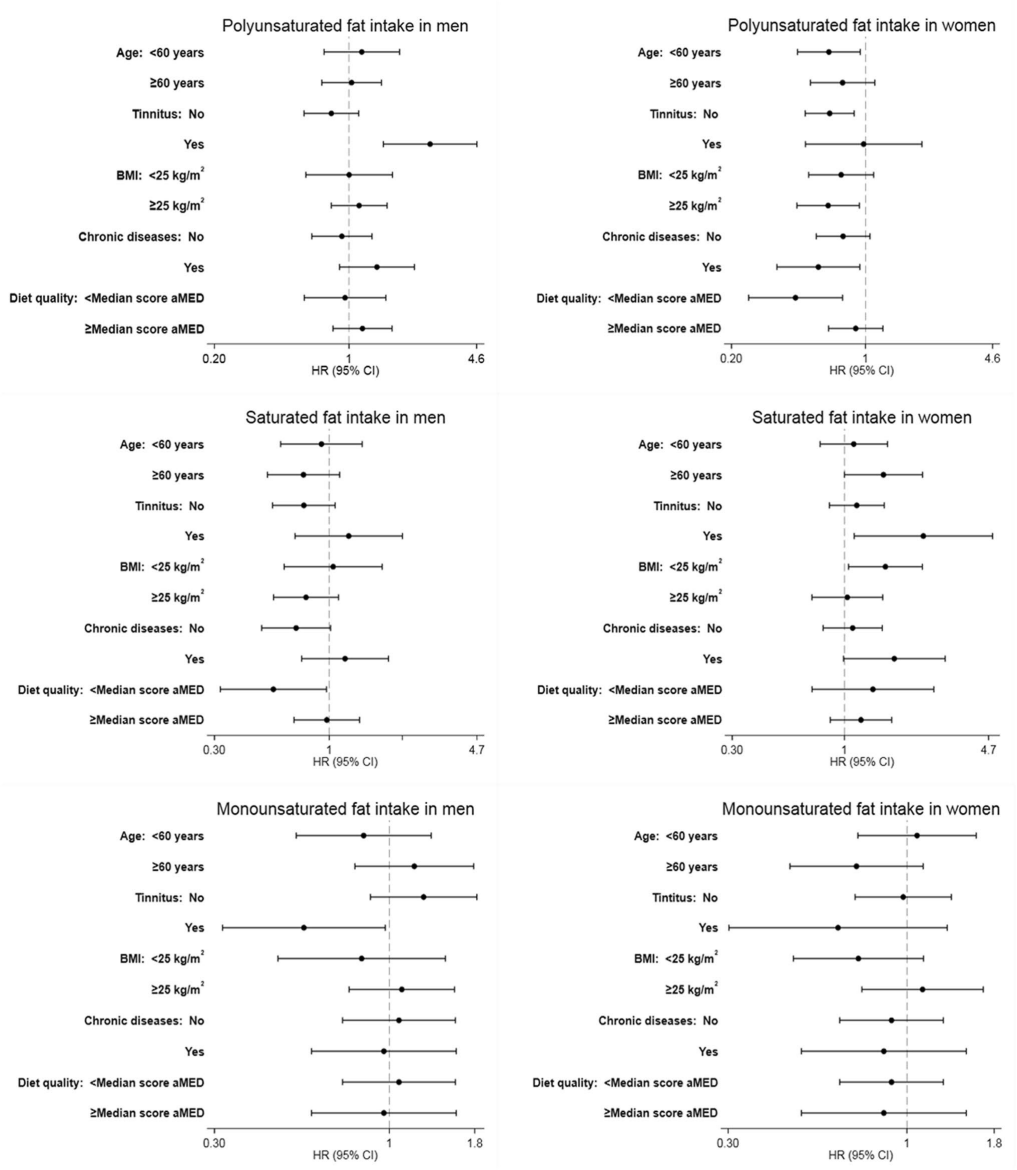
Hazard ratios (95% confidence interval) for the association between dietary fat intake and risk of disabling hearing function in the UK Biobank study stratified by sex in subgroups of participants (quintile of highest intake compared to the quintile of lowest intake). Cox regression model adjusted for age, ethnic background, educational level (≤ primary, secondary, university), tobacco (current smoker, former smoker, never smoker), BMI (< 25.0, 25.0–29.9, ≥ 30.0 kg/m^2^), physical activity (quintiles of METs-h/wk), alcohol consumption (quintiles g/d), loud music exposure (yes/no), noisy workplace (no, for less than a year, for around 1–5 years, for more than 5 years), tinnitus, aspirin, ibuprofen consumption, reaction time (ms), hypercholesterolemia, vascular/heart problems, cancer, diabetes, total energy (quintiles of kcal/day), protein intake (quintiles of % energy), and for PUFA, SFA, and MUFA (in quintiles of % energy), as appropriate

The results were similar when they were restricted to participants with optimal hearing at baseline (Supplemental Table 2). In stratified analyses, women with the highest PUFA intake and < 60 y, without tinnitus, with BMI ≥ 25 kg/m^2^, with chronic diseases, or with low adherence to a Mediterranean diet pattern had lower risk of disabling hearing impairment (*p* for interaction non-significant in any case), compared with those with the lowest intake and none of these characteristics. For SFA, we observed a higher risk for those women with the highest intake and ≥ 60 y, with tinnitus, BMI < 25 kg/m^2^ or chronic diseases (p for interaction non-significant). For MUFA, no association was found in these analyses (Fig. 2).

## Discussion

In this large population-based cohort study, we found that energy intake > 15% from PUFA was associated with lower risk of disabling hearing impairment among women. Replacing 5% of energy intake from SFA for PUFA was associated with a significant reduced risk. By contrast, no association was observed among men.

Gopinath et al. [17], using data of 2000 participants in the Blue Mountains Hearing Study, found that higher increase in n-3 PUFA was associated with reduced incident hearing loss. Hearing loss was defined as hearing impairment > 25 dB in the pure-tone average of audiometric hearing thresholds at 500, 1000, 2000, and 4000 Hz, at the 5-year follow-up examination. In addition, Curhan et al. [16] found that, among 60,000 women from the Nurses’ Health Study, those with an n-3 PUFA intake on the highest vs. lowest quintile of the distribution had a lower risk of self-reported hearing loss (RR: 0.85; 95% CI: 0.80–0.91). Both studies suggested that the plausible beneficial effect of these fats could be due to the hypolipidemic, triglyceride lowering, and anti-inflammatory and antiatherothrombotic properties of some PUFA, which help to maintain adequate vascular supply to the cochlea [43]. However, in a recent cross-sectional study, with plasma concentrations of n−3 and n−6 PUFAs measured in 534 participants, no clear link was found between plasma levels of PUFAs and hearing function, assessed using audiometric measures [44].

The causes of hearing function impairment are multiple. The main ones include the exposure to environmental risk factors, age-associated degenerative processes, and chronic diseases that alter blood supply [45]. If there is inadequate irrigation of the auditory system, cochlear function could be affected. Alterations in the vascularization of the auditory system could hinder the evacuation of waste from cellular metabolism, that leads to the development of microvascular diseases [46]. It has been observed that an increase in PUFA intake through diet could reduce pathologies related to microvascularization [42], including impaired renal function [47].

We found a statistically significant interaction between the PUFA intake and sex on the risk of disabling hearing impairment. Some studies suggest that estrogens could protect hearing function [9, 48]. There is also evidence of sex differences in the reception of complex stimuli such as speech, possibly due to differences in the activation of language processing at the cortical level, because of the distinct length of the cochlea in men and women [49]. Understanding the biological pathways that explain sex differences associated with nutritional exposures on health outcomes seems necessary to develop targeted strategies for the population [50].

Stratified analyses were robust showing a reduced risk associated with higher intake of PUFA in women. We observed stronger associations in women younger than 60 years; this is biologically plausible, since at increasing age, the ability to identify speech stimuli decreases greatly and beneficial dietary effects may be insufficient to reverse this situation [51]. We also observed a stronger inverse association among women without tinnitus. Since tinnitus and hearing loss are strongly related, and PUFA intake has also been related to tinnitus [19], it is unclear if tinnitus can partly mediate in this association. Furthermore, the very strong associations found in those with excess weight or having chronic diseases may indicate that common biological mechanisms underlie hearing impairment, obesity, and chronic diseases [39, 52].

Regarding our findings on the intake of SFA, we observed a higher risk of hearing impairment associated to consumptions above 10% of energy intake. Although we could not find a dose–response association, substitution analyses showed that replacing PUFA for SFA decreased the risk of disabling hearing loss; more research is needed to understand the role of SFA in hearing function. In addition, replacing PUFA for MUFA was also associated with a slight reduction of risk. A recent paper has suggested that MUFA from sources other than olive oil (e.g., red meats and high-fat dairy products) may have a detrimental effect on health [53].

The strengths of our study lie in its prospective design and large sample size. Besides, the hearing measurement test includes not only pure tones but also speech in noise that simultaneously scans many frequencies of human speech, especially those close to 1000 Hz. Furthermore, our analyses were adjusted for many potential confounders, such as lifestyle, exposure to high-intensity sounds, ototoxic medication, and morbidity. The study also has several limitations, including the use of 24-h-recall questionnaires to estimate habitual diet. We could not explore the separate association of different PUFA, including n−3 and n−6, or MUFA from plant vs. animal sources with hearing function due to the lack of this information in the database analyzed. Specifically, since n−6 PUFA are the main source of PUFA in most populations, including the cohort analyzed here, we cannot discard that most of the effect observed is due to this fatty acid instead of n−3 PUFA, in contrast to the previous studies. Although the role of n−6 PUFA in inflammation has been controversial, our previous findings based on the effect of these fatty acids on plasma concentration of inflammatory biomarkers [54] and others on circulating arachidonic acid levels [55] suggest that dietary n−6 PUFA do not increase inflammatory processes and may help improving endothelial function and chronic inflammation.

In conclusion, higher consumption of PUFA was associated with decreased risk of disabling hearing function in women. Replacing 5% of total energy intake from SFA by the same energy from dietary PUFA may contribute to delay hearing loss. Further research may help understanding the differences found in men and women and which types of PUFAs are contributing to this association; also, other studies should elucidate if SFA intake increases the risk of hearing loss.

## Supplementary Information

Below is the link to the electronic supplementary material.

Supplementary file1 (DOCX 22 KB)

## Data Availability

Data and materials are available if required.

## Code availability

Code is available if required.
