# Supplementary material for: Dietary fat intake and risk of disabling hearing impairment: a prospective population-based cohort study
Source: Eur J Nutr. 2021 Jul 21;61(1):231–42. doi: 10.1007/s00394-021-02644-7 (PMC8783872; doi:10.1007/s00394-021-02644-7)
Supplement: Supplementary file 1 — Supplementary file1 (DOCX 22 KB) [file 394_2021_2644_MOESM1_ESM.docx]

**Supplemental table 1.** Isocaloric substitution models for the replacement of 5% of energy intake from PUFA for carbohydrates and proteins in men and women.

|  | Women |
| --- | --- |
|  | Hazard ratio (95% confidence interval) |
| PUFA for SFA | 0.75 (0.74-0.77) |
| PUFA for MUFA | 0.97 (0.94-1.00) |
| PUFA for carbohydrates | 0.87 (0.86-0.86) |
| PUFA for total protein | 0.91 (0.88-0.95) |

**Supplemental table 2**. Association between dietary fat intake (% energy) and risk of disabling hearing function in the UK Biobank study stratified by sex in participants with optimal hearing at baseline (SRTn <-5.5 dB). (N=95,836)

|  | Quintile 1 | Quintile 2 | Quintile 3 | Quintile 4 | Quintile 5 | P Trend |
| --- | --- | --- | --- | --- | --- | --- |
| **MEN** |  |  |  |  |  |  |
| Total fat intake |  |  |  |  |  |  |
| Participants, n | 8,489 | 8,597 | 8,596 | 8,630 | 8,658 |  |
| Range, % energy | 1.6-26.7 | 26.8-30.8 | 30.9-34.1 | 34.2-38.0 | 38.1-68.6 |  |
| Person-yr | 32,574 | 34,229 | 34,374 | 34,362 | 33,872 |  |
| Cases, n | 132 | 157 | 170 | 138 | 123 |  |
| Age-adjusted model | 1.00 | 0.99 (0.78-1.24) | 1.05 (0.84-1.32) | 0.88 (0.69-1.11) | 0.87 (0.68-1.12) | 0.17 |
| MV-adjusted model | 1.00 | 0.98 (0.77-1.24) | 1.04 (0.82-1.32) | 0.88 (0.68-1.13) | 0.85 (0.65-1.10) | 0.14 |
| PUFA intake |  |  |  |  |  |  |
| Participants, n | 8,524 | 8,611 | 8,616 | 8,593 | 8,626 |  |
| Range, % energy | 0.1-3.9 | 4.0-5.1 | 5.2-6.2 | 6.3-7.6 | 7.7-18.7 |  |
| Person-yr | 32,459 | 34,406 | 34,558 | 34,394 | 33,595 |  |
| Cases, n | 106 | 161 | 158 | 171 | 124 |  |
| Age-adjusted model | 1.00 | 1.22 (0.95-1.55) | 1.19 (0.93-1.52) | 1.30 (1.02-1.65) | 1.11 (0.86-1.44) | 0.46 |
| MV-adjusted model | 1.00 | 1.22 (0.95-1.58) | 1.21 (0.93-1.57) | 1.29 (0.98-1.69) | 1.08 (0.80-1.46) | 0.78 |
| SFA intake |  |  |  |  |  |  |
| Participants, n | 8,520 | 8,569 | 8,629 | 8,653 | 8,599 |  |
| Range, % energy | 0.3-9.5 | 9.6-11.5 | 11.6-13.2 | 13.3-15.2 | 15.3-30.5 |  |
| Person-yr | 33,081 | 33,802 | 34,506 | 34,416 | 33,607 |  |
| Cases, n | 145 | 161 | 146 | 152 | 116 |  |
| Age-adjusted model | 1.00 | 1.02 (0.81-1.27) | 0.86 (0.69-1.09) | 0.94 (0.75-1.18) | 0.78 (0.61-1.00) | 0.04 |
| MV-adjusted model | 1.00 | 1.04 (0.82-1.32) | 0.86 (0.67-1.11) | 0.94 (0.72-1.23) | 0.77 (0.57-1.04) | 0.07 |
| MUFA intake |  |  |  |  |  |  |
| Participants, n | 8,525 | 8,544 | 8,601 | 8,633 | 8,667 |  |
| Range, % energy | 0.6-11.3 | 11.4-13.1 | 13.2-14.7 | 14.8-16.5 | 16.6-37.0 |  |
| Person-yr | 32,590 | 34,057 | 34,344 | 34,342 | 34,079 |  |
| Cases, n | 127 | 159 | 156 | 139 | 139 |  |
| Age-adjusted model | 1.00 | 1.01 (0.80-1.28) | 0.97 (0.77-1.23) | 0.91 (0.71-1.15) | 0.96 (0.75-1.22) | 0.50 |
| MV-adjusted model | 1.00 | 0.96 (0.75-1.24) | 0.95 (0.73-1.25) | 0.91 (0.68-1.23) | 0.98 (0.71-1.36) | 0.87 |
| **WOMEN** |  |  |  |  |  |  |
| Total fat intake |  |  |  |  |  |  |
| Participants, n | 10,487 | 10,554 | 10,595 | 10,620 | 10,610 |  |
| Range, % energy | 1.6-27.2 | 27.3-31.3 | 31.4-34.6 | 34.7-38.5 | 38.5-70.9 |  |
| Person-yr | 39,009 | 40,425 | 40,599 | 40,899 | 40,084 |  |
| Cases, n | 133 | 167 | 151 | 169 | 135 |  |
| Age-adjusted model | 1.00 | 1.06 (0.84-1.33) | 0.96 (0.76-1.21) | 1.06 (0.84-1.33) | 0.95 (0.74-1.20) | 0.67 |
| MV-adjusted model | 1.00 | 1.03 (0.82-1.30) | 0.92 (0.72-1.17) | 1.00 (0.79-1.27) | 0.88 (0.69-1.13) | 0.32 |
| PUFA intake |  |  |  |  |  |  |
| Participants, n | 10,502 | 10,570 | 10,573 | 10,620 | 10,601 |  |
| Range, % energy | 0.03-4.1 | 4.2-5.3 | 5.4-6.4 | 6.5-7.9 | 8.0-25.3 |  |
| Person-yr | 37,180 | 38,750 | 38,818 | 38,762 | 38,553 |  |
| Cases, n | 143 | 158 | 161 | 185 | 119 |  |
| Age-adjusted model | 1.00 | 0.88 (0.70-1.09) | 0.82 (0.66-1.03) | 0.98 (0.78-1.22) | 0.67 (0.53-0.85) | 0.009 |
| MV-adjusted model | 1.00 | 0.85 (0.67-1.07) | 0.79 (0.62-1.01) | 0.96 (0.75-1.23) | 0.68 (0.51-0.91) | 0.05 |
| SFA intake |  |  |  |  |  |  |
| Participants, n | 10,475 | 10,561 | 10,640 | 10,605 | 10,585 |  |
| Range, % energy | 0.1-9.6 | 9.7-11.5 | 11.6-13.2 | 13.3-15.3 | 15.4-33.8 |  |
| Person-yr | 38,782 | 40,396 | 40,940 | 40,741 | 40,157 |  |
| Cases, n | 110 | 171 | 163 | 161 | 150 |  |
| Age-adjusted model | 1.00 | 1.31 (1.03-1.66) | 1.21 (0.95-1.54) | 1.23 (0.96-1.56) | 1.22 (0.96-1.56) | 0.27 |
| MV-adjusted model | 1.00 | 1.32 (1.03-1.69) | 1.23 (0.94-1.59) | 1.20 (0.92-1.57) | 1.24 (0.92-1.65) | 0.40 |
| MUFA intake |  |  |  |  |  |  |
| Participants, n | 10,466 | 10,585 | 10,572 | 10,628 | 10,615 |  |
| Range, % energy | 0.83-11.5 | 11.6-13.3 | 13.4-14.9 | 15.0-16.8 | 16.9-38.7 |  |
| Person-yr | 38,953 | 40,578 | 40,705 | 40,909 | 39,871 |  |
| Cases, n | 136 | 166 | 172 | 163 | 118 |  |
| Age-adjusted model | 1.00 | 1.04 (0.83-1.30) | 1.05 (0.84-1.32) | 1.00 (0.79-1.25) | 0.83 (0.65-1.06) | 0.13 |
| MV-adjusted model | 1.00 | 1.05 (0.82-1.34) | 1.05 (0.81-1.35) | 0.98 (0.74-1.30) | 0.83 (0.60-1.14) | 0.20 |

PUFA: polyunsaturated fatty acids; SFA: saturated fatty acids, MUFA: monounsaturated fatty acids.

Values are hazard ratios (95% confidence interval).

Multivariable (MV) adjusted model: Cox regression model adjusted for age, ethnic background, educational level (≤ primary, secondary, university) tobacco (current smoker, former smoker, never smoker), BMI (<25.0, 25.0-29.9, ≥30.0 kg/m^2^), physical activity (quintiles of METs-h/wk), alcohol consumption (quintiles of g/d), loud music exposure (yes/no), noisy workplace (no, for less than a year, for around 1-5 years, for more than 5 years), tinnitus, aspirin, ibuprofen consumption, reaction time (msec), hypercholesterolemia, vascular/heart problems, cancer, diabetes, total energy (quintiles of kcal/day), and protein intake (quintiles of % energy). Models for PUFA were adjusted for SFA and MUFA and vice versa (quintiles of % energy).
